# NMR Relaxation to Probe Zeolites: Mobility of Adsorbed Molecules, Surface Acidity, Pore Size Distribution and Connectivity

**DOI:** 10.3390/molecules29225432

**Published:** 2024-11-18

**Authors:** Marina G. Shelyapina

**Affiliations:** Department of Nuclear Physics Research Methods, Saint Petersburg State University, 7/9 Universitetskaya nab., Saint Petersburg 199034, Russia; marina.shelyapina@spbu.ru

**Keywords:** nuclear magnetic resonance, spin relaxation, zeolites, microporous materials, mesoporous materials, hierarchical porosity, Lewis acid sites, Brønsted acid sites, diffusion, NMR cryoporometry, pore connectivity

## Abstract

Unique structural and chemical properties, such as ion exchange, developed inner surface, etc., as well as the wide possibilities and flexibility of regulating these properties, cause a keen interest in zeolites. They are widely used in industry as molecular sieves, ion exchangers and catalysts. Current trends in the development of zeolite-based catalysts include the adaptation of their cationic composition, acidity and porosity for a specific catalytic process. Recent studies have shown that mesoporosity is beneficial to the rational design of catalysts with controlled product selectivity and an improved catalyst lifetime due to its efficient mass-transport properties. Nuclear magnetic resonance (NMR) has proven to be a reliable method for studying zeolites. Solid-state NMR spectroscopy allows for the quantification of both Lewis and Brønsted acidity in zeolite catalysts and, nowadays, ^27^Al and ^29^Si magic angle spinning NMR spectroscopy has become firmly established in the set of approved methods for characterizing zeolites. The use of probe molecules opens up the possibility for the indirect measurement of the characteristics of acid sites. NMR relaxation is less common, although it is especially informative and enlightening for studying the mobility of guest molecules in the porous matrix. Moreover, the NMR relaxation of guest molecules and NMR cryoporometry can quantify pore size distribution on a broader scale (compared to traditional methods), which is especially important for systems with complex pore organization. Over the last few years, there has been a growing interest in the use of 2D NMR relaxation techniques to probe porous catalysts, such as 2D $T_{1}$–$T_{2}$ correlation to study the acidity of the surface of catalysts and 2D $T_{2}$–$T_{2}$ exchange to study pore connectivity. This contribution provides a comprehensive review of various NMR relaxation techniques for studying porous media and recent results of their applications in probing micro- and mesoporous zeolites, mainly focused on the mobility of adsorbed molecules, the acidity of the zeolite surface and the pore size distribution and connectivity of zeolites with hierarchical porosity.

## 1. Introduction

**Figure 1:**
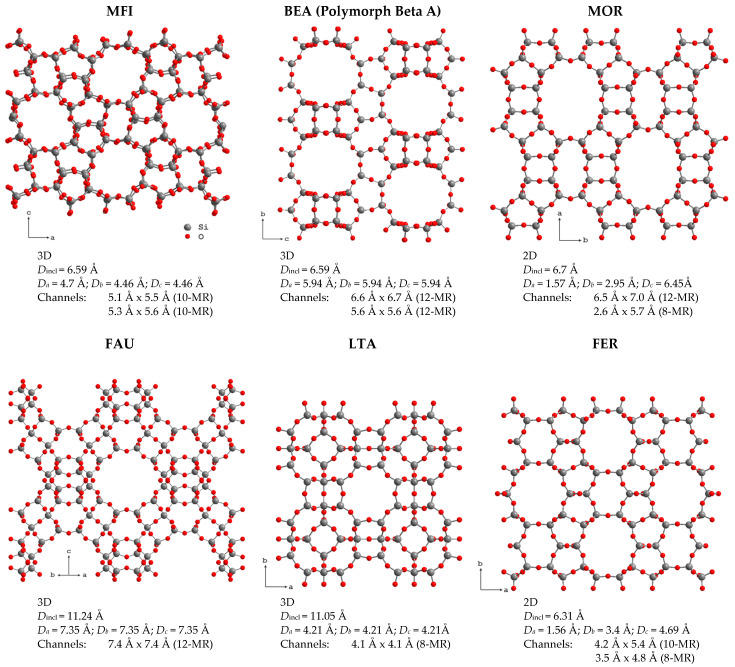
Selected zeolite frameworks and information about their pore structure: channel dimensionality; the maximum diameter of a sphere that can be included (*D*_incl_) and that diffuses in a specific direction *x* (*D_x_*); the channel size and the *N*-membered ring it is formed by (in parenthesis).

Zeolites form a large group of crystalline aluminosilicates. Their crystal structures can be represented as open three-dimensional frameworks assembled from [SiO_4_]^4−^ and [AlO_4_]^5−^ tetrahedra, connected by vertices that form regular cavities and channel systems in one, two or three dimensions depending on the zeolite framework topology. At the moment, 256 different topologies of the zeolite framework are known [1]. Selected zeolite structures and brief information about their channels, taken from the International Zeolite Association database [1], are shown in Figure 1.

The size of the formed voids varies from 0.3 to 1.0 nm. This means that, according to the International Union of Pure and Applied Chemistry (IUPAC), 3D zeolites are microporous materials (the IUPAC classification of porous materials according to their pore sizes *d* is as follows: microporous if d<2 nm, mesoporous if 2<d<50 nm and macroporous if d>50 nm). Depending on the framework type, zeolites can be stable in different ranges of the Si/Al ratio. The substitution of Al^3+^ for Si^4+^ results in the negative charge of the zeolite framework that is compensated by extra-framework cations, such as Na^+^, K^+^, NH_4_^+^, Ca^2+^, etc., located in the zeolite voids, or protons that form various OH groups on the zeolite inner surface [2].

The unique structural and chemical properties of zeolites, such as ion exchange, their developed inner surface and their ordered pore structure, as well as the wide possibilities and flexibility of regulating these properties either during synthesis or as a result of post-synthetic processing, cause a wide interest in zeolites and, nowadays, this interest is only increasing, especially in view of the accepted strategies for sustainable development and the transition to resource-saving energy [3]. It should be noted that zeolites are quite common minerals and are widely used in industry as molecular sieves and ion exchangers [4]. Another important application of zeolites that is demanded in the market is catalysis. Until recently, synthetic zeolites have dominated in this field [5,6]. Current trends in the development of zeolite-based catalysts include the adaptation of their cationic composition [4,7,8,9], porosity [10,11,12] and acidity [13,14,15] for specific catalytic processes.

Synthetic ZSM-5 (MFI), beta-zeolite (BEA), mordenite (MOR), zeolite Y (FAU) and ferrierite (FER) form the so-called “big five” zeolites, which are widely used in industry as catalysts [16]. The main reasons for their use are their unique pore structure, which ensures a perfect match with the requirements of the target reactions, and the presence of active sites and accessibility of these sites to guest molecules [17]. Brønsted and Lewis acid sites play key roles in the efficiency and selectivity of many catalytic processes. The reactivity of these acid sites is determined by the density, spatial arrangement and local environment of the aluminum atoms in the zeolite framework. In addition, ion exchange [4,7,18,19], the incorporation of metallic nanoparticles [8,20,21,22] or oxo-cation clusters [23,24,25,26] and isomorphous substitution with the introduction of different heteroelements [8,27,28] into zeolites allows for the tailoring of the nature and catalytic function of the formed acid sites.

At the same time, the transport properties of the zeolite matrix, as well as the type and strength of the interaction of the adsorbed molecules with the inner surface of the zeolite, are of great importance [29,30]. Recent studies have shown that mesoporosity is beneficial for the rational design of catalysts with controlled product selectivity [31,32] and helps to improve the catalyst’s lifetime [33,34] due to its efficient mass-transport properties.

To tailor the structure and properties of zeolite-based catalysts for specific applications, a comprehensive study of their physicochemical properties is necessary. Nuclear magnetic resonance (NMR) has proven to be a reliable method for studying zeolites [35,36,37,38]. Magic angle spinning (MAS) NMR on the ^29^Si and ^27^Al nuclei allows for the quantification of the silica to alumina ratio in the material, which is considered to be an analog of the acidity of the zeolite [13,39]. Moreover, ^27^Al MAS NMR enables the aluminum that is a part of the zeolite framework (tetra-co-ordinated Al) to be distinguished from the disordered, so-called penta-co-ordinated Al, and the extra-framework Al species (six-co-ordinated Al) [40,41]; the penta- and six-co-ordinated species, in turn, are issues of Brønsted [42] and Lewis acidity [39,43], respectively. Solid-state ^1^H MAS NMR spectroscopy provides a direct quantitative determination of the density of Brønsted acid sites [44], distinguishing the bridge Si–OH–Al and terminal Al–OH or Si–OH groups from the observed ^1^H chemical shift.

The application of probe molecules opens up the possibility of indirect studying of the zeolite surface acid sites and its pore structure. In particular, ^1^H chemical shift can be used for detailed study of the interactions of adsorbed water molecules with the zeolite surface [45]. In the case of organic probe molecules (such as phosphine oxides [46,47,48,49], pyridine [50,51], acetone [52,53], carbon dioxide [54], etc.), a wide range of experiments involving ^31^P, ^15^N and ^13^C MAS NMR are successfully applied to study zeolite acidity.

^129^Xe NMR is another powerful tool for determining both the porosity and pore connectivity of zeolites [55,56,57]. Being sensitive to the chemical composition and the physical structure of its environment, ^129^Xe is an ideal probe gas for NMR. However, in practice, for better sensitivity, ^129^Xe has to be hyperpolarized via spin exchange optical pumping (SEOP) [58,59], dynamic nuclear polarization (DNP) [60] or by the so-called brute force method in high magnetic fields and very low temperatures (on the order of millikelvins) [61].

In recent years, zero- to ultralow-field, below 10 μT, NMR have appeared. In contrast to conventional high-field NMR, in ultralow-field NMR experiments, chemical shifts vanish and spectra are governed by indirect nuclear spin–spin couplings [62]. Without magnetic field inhomogeneity, the high resolved spectra enable monitoring of the chemical reaction in the liquid phase [63]. Several studies demonstrate that ultralow-field NMR is a promising tool for probing liquid–surface adsorption dynamics of molecules inside porous materials [64,65]; however, there have been few such studies.

It should be noted that the vast majority of NMR studies of zeolites are focused on NMR spectroscopy; meanwhile, another NMR technic, relaxation, is especially informative for studying the mobility of guest molecules confined in pores [45,66,67,68,69]. Over the last few years, there has been a growing interest in the use of NMR relaxation in a relatively low magnetic field to probe the surface acidity of mirco- and mesoporous catalysts [70,71,72], pore size distribution [73,74,75] and pore connectivity [74,76]; the latter is especially valuable for systems with hierarchical porosity.

This review presents various NMR relaxation techniques for probing zeolites, both traditional methods for studying the mobility of adsorbed molecules via relaxation time measurements (including diffusometry) and, less frequent in the past but becoming more and more popular, NMR cryoporometry, 2D $T_{1}$–$T_{2}$ correlation and 2D $T_{2}$–$T_{2}$ exchange experiments.

## 2. Basic NMR Pulse Sequences for Relaxation Measurements

Before discussing application to zeolites, let us provide the basic concepts of NMR relaxation and describe the main pulse sequences for determining the nuclear relaxation times.

Let us consider a sample consisting of nuclei with spin *I* > 0 and, therefore, having magnetic moment. In a constant magnetic field ${\vec{B}}_{0}$ (let us suppose it is along the *z*-axis of the laboratory frame), the nuclear magnetization $\vec{M}$ (the sum of all nuclear spins) is in the equilibrium state and parallel to the magnetic field ${\vec{B}}_{0}$. If one applies a radiofrequency (rf) pulse, which is oscillating at or near to the so-called Larmor frequency $\omega_{0}=\gamma B_{0}$ (γ is a gyromagnetic ratio for the nuclei under study) and perpendicular to ${\vec{B}}_{0}$, for example, along the *x*-axis of the laboratory frame, it turns the nuclear magnetization around this axis. The pulse duration and amplitude determine the deviation angle of the nuclear magnetization from its equilibrium state: 90° pulse means that it turns the nuclear magnetization in a plane perpendicular to the *z*-axis; 180° pulse means that it inverts the nuclear magnetization. After the rf pulse is switched off, the nuclear magnetization relaxes to its equilibrium state, wherein the recovery of the longitudinal component of the nuclear magnetization (parallel to ${\vec{B}}_{0}$) and the disappearance of the transverse component (perpendicular to ${\vec{B}}_{0}$) obey exponential laws but with different characteristic times: spin–lattice ($T_{1}$) and spin–spin ($T_{2}$) relaxation times, respectively.

The issues of these relaxation processes are interactions of the nuclear spin with neighboring spins and with fluctuating local electric and magnetic fields. More details on the theoretical background of NMR relaxation can be found anywhere [77]. The values of $T_{1}$ and $T_{2}$ for a specific nucleus, their temperature and/or frequency dependences characterize both the local structure and dynamics of the system under study.

### 2.1. $T_{1}$ and $T_{1\rho }$ Measurements

**Figure 2:**
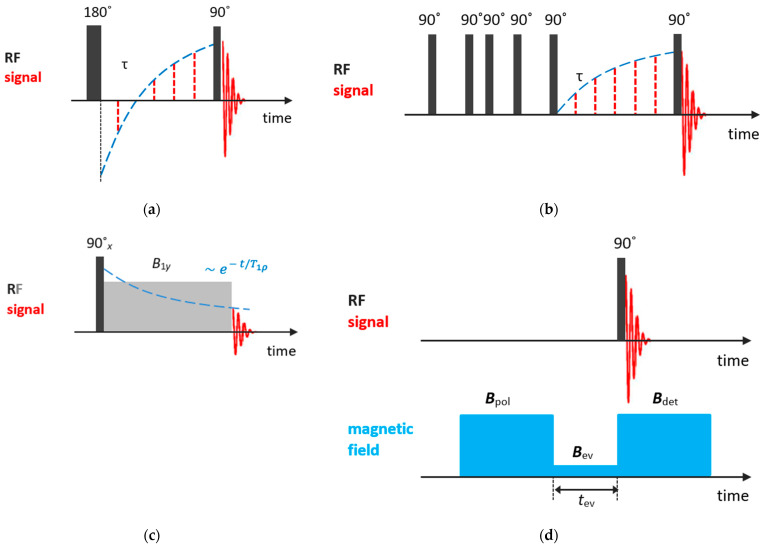
Basic NMR sequences for $T_{1}$ and $T_{1\rho }$ measurements: (**a**) inversion-recovery; (**b**) saturation-recovery; (**c**) spin-locking; (**d**) field cycling.

Among methods for measuring spin–lattice relaxation times $T_{1}$, the most popular are inversion-recovery and saturation-recovery pulse sequences. The inversion-recovery method comprises a 180° pulse, inverting the nuclear magnetization $\vec{M}$, followed by a 90° pulse in time τ to measure the recovering magnetization. For the recovery time, the magnetization starts out inverted and, with τ increasing, passes through zero, recovering its equilibrium value $M_{0}$; see Figure 2a. The evolution of the *z*-projected nuclear magnetization is described by the following equation:

(1) $$M_{z}\left(\tau \right)=M_{0}\left[1-2\exp\left(-\frac{\tau }{T_{1}}\right)\right].$$

However, it should be noted that, before applying the 180° pulse, the spin system must be in equilibrium that is achieved at ${t>5T}_{1}$. For systems with long $T_{1}$, this method can be rather time-consuming.

In the saturation-recovery experiment, Figure 2b, two 90° pulses separated by τ are applied. The first pulse destroys the nuclear magnetization and the signal measured after the second pulse is described by:

(2) $$M_{z}\left(\tau \right)=M_{0}\left[1-\exp\left(-\frac{\tau }{T_{1}}\right)\right].$$

In this method, the requirement for the equilibrium of the nuclear magnetization before the first pulse is not so strict and, therefore, this method is more time-saving.

Actually, there are many other methods for determining $T_{1}$; the choice between them depends on the studied system or process and on the NMR equipment available to a researcher. For systems with long $T_{1}$ and weak NMR signal, the progressive saturation method, which is closely related to the saturation-recovery experiment, can be used [78]. For a quantitative NMR analysis, widely used in organic chemistry in those areas where an express analysis is required (e.g., in reaction monitoring, mechanistic analysis and purity determination), for the estimation of $T_{1}$, a newly proposed Faster Longitudinal relaxation Investigated by Progressive Saturation (FLIPS) is a good choice [79]. More details concerning a comparative analysis between various techniques for measuring $T_{1}$ can be found in Ref. [80].

As will be discussed further, relaxation times are temperature- and frequency-dependent. From the temperature dependence of relaxation times, especially $T_{1}$, one can determine parameters of molecular motion. However, for systems with relatively slow molecular dynamics, such as liquids confined in zeolite voids, more informative is the so-called relaxation time $T_{1\rho }$, which characterizes the equilibration of the nuclear magnetization along the rf field ${\vec{B}}_{1}$ in the rotating frame. $T_{1\rho }$ can be measured by applying the spin-locking techniques. The experiment involves three stages; see Figure 2c. First, the nuclear magnetization is oriented by the rf field $B_{1x}$ (usually by applying a 90° pulse). Second, the spin system evolves in the rf field $B_{1y}$ (the locking pulse that keeps the magnetization along the *y*-axis), with the characteristic relaxation time $T_{1\rho }$. Finally, after the magnetic field $B_{1y}$ is switched off, the rest nuclear magnetization is recorded. For more details, see Ref. [77].

Frequency dependences of relaxation times provide additional information on molecular motion; however, usually only fixed-frequency NMR spectrometers are available. Nevertheless, it can be accessed through a field cycling relaxometry that requires special settings [81].

In the field cycling experiment, the magnetic field is periodically switched; Figure 2d. First, to attain the equilibrium magnetization of nuclear spins, a high polarization field ${\vec{B}}_{pol}$ is applied. Second, the magnetic field is quickly switched to a variable evolution field ${\vec{B}}_{ev}$, to allow the nuclear magnetization to relax toward its new equilibrium value. To trace this equilibration, the magnetic field is quickly switched back to a suitable detection field ${\vec{B}}_{det}$. Finally, to register the nuclear magnetization at a given ${\vec{B}}_{ev}$ and evolution time $t_{ev}$, a 90° pulse is applied. This field cycle is repeated for various evolution times and evolution fields to store the frequency-dependent magnetization decay. A more detailed description can be found in Ref. [81].

### 2.2. $T_{2}$ Measurements

**Figure 3:**
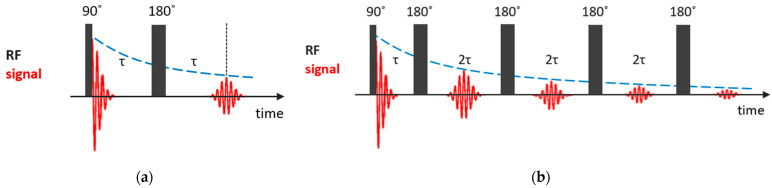
Basic NMR sequences for $T_{2}$ measurements: (**a**) Hahn spin echo; (**b**) CPMG pulse train.

The spin–spin relaxation time $T_{2}$ quantifies the rate of the decay of the transversal component of the nuclear magnetization recovering to its equilibrium state. If one neglects the inhomogeneity of the magnetic field, the simplest way to measure the $T_{2}$ time is to record the free induction decay (FID) signal after a 90° pulse. However, under the condition of magnetic field inhomogeneity, which is always the case, the measured value, $T_{2}^{*}$, is shorter than the real $T_{2}$ value, and additional efforts are needed to determine spin–spin relaxation times. The simplest way to measure it is the Hahn spin echo (SE) pulse sequence [82], in which, after the first 90° pulse, after a time of τ, during which the dephasing of nuclear magnetization precessions occurs due to the magnetic field inhomogeneity, a 180° pulse follows and, after another time of τ, the precession is phased and a spin echo signal appears; see Figure 3a. The echo amplitude at time 2τ is given by the simple expression:

(3) $$S^{SE}\left(2\tau \right)=S_{0}\exp\left(-\frac{2\tau }{T_{2}}\right).$$

In this method, the requirement of the equilibrium for the nuclear magnetization before the first pulse is not so strict. However, in the presence of translational diffusion, an additional contribution to the echo decay appears. The effect of diffusion can be suppressed by applying a Carr–Purcell–Meiboom–Gill (CPMG) pulse train [83,84], in which a series of 180° pulses follows after a two-pulse Han echo sequence at intervals of 2τ; see Figure 3b. To compensate imperfections in the 180° pulses, the subsequent 180° pulses are shifted in phase by 90° relative to the first 90° pulse. The amplitude of the echo signal at times 2τn is described by the following expression:

(4) $$S^{CPMG}\left(2\tau n\right)=S_{0}\exp\left(-\frac{2\tau n}{T_{2}}\right)\exp\left(-\frac{D\gamma^{2}G_{0}^{2}{\left(2\tau \right)}^{3}n}{3}\right),$$

where D is the diffusion coefficient and $G_{0}$ is the magnetic field gradient. By decreasing τ, one can make the diffusional decay arbitrarily small. Currently, CPMG is a fundamental component of pulse sequences used in NMR to study dynamic processes.

### 2.3. Self-Diffusion Measurements

**Figure 4:**
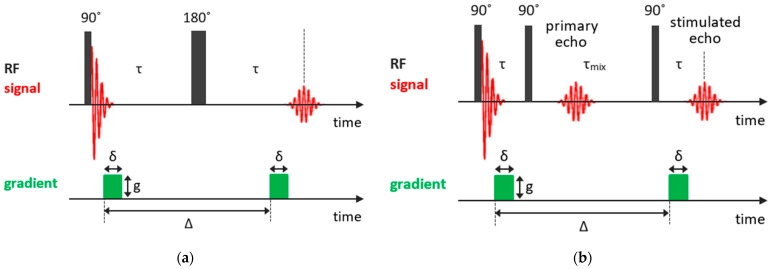
Basic NMR sequences for diffusion measurements: (**a**) PGSE experiment; (**b**) PFG STE experiment.

As was mentioned previously, in the Hahn echo experiment, molecular diffusion results in spin echo attenuation (it should be noted that, in NMR, one normally deals with self-diffusion, which is often referred to simply as diffusion). Thus, a gradient-assisted experiment can be designed in such a way to explore the diffusion coefficients. In the pulsed gradient spin echo (PGSE) method [85], which is based on the Hahn spin echo sequence, two gradient pulses g of equal phase and length are applied for spatially encoding the nuclear spins along the gradient direction. The first gradient pulse follows the 90° pulse after a short delay $t_{1}$ and the second gradient pulse is applied after the 180° rf pulse with a time interval between the two gradient pulses Δ (the diffusion delay) and acts to spatially decode the spins in the sample. The PGSE pulse sequence is shown in Figure 4a. This technique of the spatially encoding nuclei with the magnetic field gradient extended to three dimensions is used in magnetic resonance imaging.

For systems with short $T_{2}$, which is often the case of solids or confined liquids, more effective is the stimulated echo (STE) pulse sequence, in which the 180° pulse is split into two 90° pulses. So, the STE pulse sequence consists of three 90° rf pulses: the first 90° pulse creates the transverse nuclear magnetization, which begins to out-phase; the second 90° pulse applied in time τ turns the out-phased magnetization along the *z*-axis and, during the mixing time $t_{m}\gg \tau$, the magnetization decays due to both the spin-lattice relaxation and diffusion in the field gradient; finally, the third 90° pulse in time $t_{m}+\tau$ transforms the magnetization again to the transverse one; and, at time $t_{m}+2\tau$, an echo signal appears. Similar to the PGSE experiment, the transverse magnetization is coded by the first gradient pulse and then decoded by the second pulse after a delay, during which diffusion occurs. The magnetic field gradient pulses of amplitude g and duration δ are applied after the first and third 90° pulses, with an interval between gradient pulses Δ. The pulsed-field gradient stimulated echo (PFG STE) experiment is shown in Figure 4b. The dependence of the echo signal attenuation is expressed as follows:

(5) $$S^{PGF-STE}\left(t_{m},\tau ,g\right)=S_{0}\exp\left(-\frac{2\tau }{T_{2}}\right)\exp\left(-\frac{t_{m}}{T_{1}}\right)\exp\left(-\frac{\gamma^{2}g^{2}\delta^{2}D(\Delta -\delta /3)}{3}\right).$$

It should be noted that gradient pulses induce eddy currents that distort the recorded signal. These currents must be allowed to dissipate before the FID acquisition. There are several strategies to take it into account, for example, the longitudinal eddy-corrected delay (LED) experiment [86] that can be further extended to the bipolar pulse LED (BPPLED) method [87]. However, these problems are more acute for magnetic resonance imaging and will not be discussed here.

The PFG NMR sequence can be combined with various 2D NMR techniques to benefit from additional analytical dimension (3D diffusion-ordered spectroscopy (DOSY) experiments), but it is beyond the present review; more information can be found anywhere [88].

As it follows from Equation (5), for a given mean molecular displacement, the signal attenuation becomes more essential with increasing intensity and duration of the gradient pulse. For systems with relatively slow molecular diffusion, the static field gradient (SFG) experiment can be more effective [89]. The observed echo amplitude in this case can be described by the following expression:

(6) $$S^{SGF-STE}\left(t_{m},\tau ,g\right)=S_{0}\exp\left(-\frac{2\tau }{T_{2}}\right)\exp\left(-\frac{t_{m}}{T_{1}}\right)\exp\left(-\gamma^{2}g^{2}D\left(\frac{2}{3}\tau^{3}+\tau^{2}t_{m}\right)\right).$$

As one can see from Equations (5) and (6), to determine the diffusion coefficient, one needs to know both *T*_1_ and *T*_2_, which can be determined by applying the methods described above.

### 2.4. $T_{1}$–$T_{2}$ Correlation and $T_{2}$–$T_{2}$ Exchange Experiments

Recently, $T_{1}$–$T_{2}$ correlation and $T_{2}$–$T_{2}$ exchange experiments became popular for characterizing the pore structure and functionality of porous materials [90]. On the one hand, this is explained by the rather modest requirements for the NMR equipment and, on the other, by the development of numerical processing methods.

**Figure 5:**
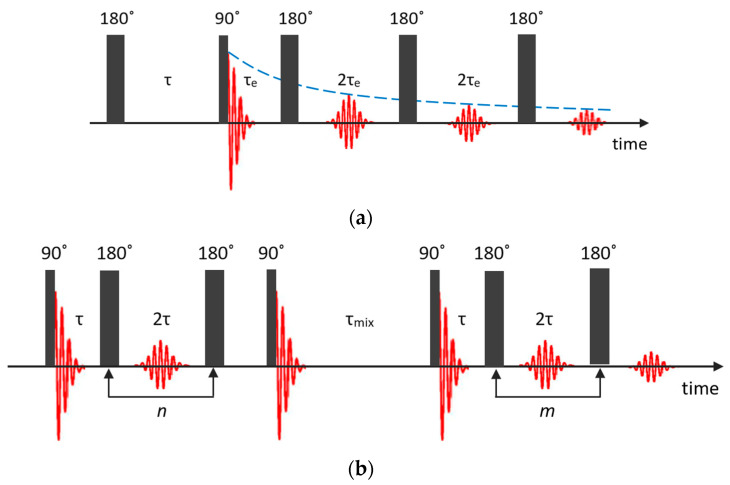
Pulse sequences used for the $T_{1}$–$T_{2}$ correlation (**a**) and $T_{2}$–$T_{2}$ exchange (**b**) experiments.

In the $T_{1}$–$T_{2}$ correlation experiment, data can be acquired using an NMR pulse sequence, shown in Figure 5a, which is composed of an inversion recovery pulse sequence followed by a CPMG echo train [91]. The recovery time τ and the echo time $\tau_{e}$ are two independent variables.

The acquired 2D NMR relaxation data, which are the normalized spin echo magnitudes, may be described by the following expression through the Fredholm integral equation of the first kind [91,92]:

(7) $$\frac{{S(\tau ,n\tau }_{e})}{S(\tau \to \infty ,0)}=\iint exp\left(\frac{-{n\tau }_{e}}{T_{2}}\right)\;\left[1-2exp\left(\frac{-\tau }{T_{1}}\right)\right]F(T_{1},T_{2})dT_{1}dT_{2}+E{(\tau ,n\tau }_{e}),$$

where $F(T_{1},T_{2})$ represents the desired 2D distributions of $T_{1}$ and $T_{2}$ relaxation times and $E{(\tau ,n\tau }_{e})$ is the experimental noise. The data analysis relies on inversion of the integral to extract *F*(*T*_1_, *T*_2_) from the measured signal amplitude.

The $T_{2}$–$T_{2}$ exchange experiment comprises two CPMG echo trains *A* and *B*, during which the spin–spin relaxation first evolves and afterwards is detected [93,94]; see Figure 5b. These two echo trains are separated by a mixing period $\tau_{mix}$ that is often referred to as the storage interval. The acquired magnetization from two isolated populations can be written as follows [95]:

(8) $$\frac{{S(t_{A},t_{B},\tau }_{mix})}{S(\tau \to \infty ,0)}=\iint exp\left(\frac{-t_{A}}{T_{2}^{A}}\right)exp\left(\frac{-t_{B}}{T_{2}^{B}}\right)\;F(T_{2}^{A},T_{2}^{B},\tau_{mix})dT_{2}^{A}dT_{2}^{B}+E(t_{A},t_{B}),$$

where $F(T_{2}^{A},T_{2}^{B},\tau_{mix})$ is the probability density of the signal components with the relaxation times $T_{2}^{A}$ and $T_{2}^{B}$, $E(t_{A},t_{B})$ is the experimental noise, and $t_{A}=n\tau$ and $t_{B}=m\tau$ are the relaxation-encoding time intervals that correspond to the durations of two CPMG echo trains. During the mixing period, nuclear magnetization relaxes with a characteristic time $T_{1}$ to its thermodynamic equilibrium along the external magnetic field. Therefore, exchange of magnetization from one site to another must be considered during all the three periods $t_{A}$, $t_{B}$ and $\tau_{mix}$ [96] and the total signal amplitude is reduced by $exp\left(-\tau_{mix}/T_{1}^{AB}\right)$.

On the $T_{2}$–$T_{2}$ map, the majority of the signal lies on the diagonal line $T_{2}^{A}=T_{2}^{B}$. An exchange between regions with different $T_{2}$ results in the appearance of symmetric pairs of off-diagonal peaks, whose intensity relative to the main peaks quantifies the exchange occurring within the mixing time.

## 3. NMR Relaxation and Diffusometry to Study Molecular Dynamics in Zeolites

The details of the dynamics of molecules adsorbed in zeolite pores govern most of the properties of these substances, which are important for applications. As was mentioned above, the selectivity of the catalytic reaction is determined by the geometry of the zeolite cavities, among others, since it affects the mechanism of the molecule reorientation. The interactions of adsorbed molecules with charge-compensating cations, hydroxyls of different natures or zeolite framework defects restrict the mobility of molecules confined in zeolite cavities and indirectly impact the overall catalytic activity of the material. And NMR, whose parameters are sensitive to fluctuations in local magnetic and electric fields at the nuclear sites due to reorientational and translational motion of molecules, provides a unique tool to probe the dynamics of adsorbed molecules in a wide range of jump rates $\tau_{c}^{-1}$ from 10^4^ to 10^11^ s^−1^ that can be expanded up to 10^−1^ s^−1^ to probe slow motions in solids when applying special techniques [97,98].

### 3.1. Nuclear Dipole Relaxation and Bloembergen–Purcell–Pound Model

For many of the systems considered here, the main issues of NMR relaxation are fluctuating strengths of nuclear dipole coupling. Being dependent on the relative position of the interacting nuclear spins, these interactions are altered by atomic motion that creates fluctuations in the magnetic field at nuclei and, therefore, fluctuations in the Larmor frequency Δω [77]. This process can be described in terms of a correlation function G(t) that contains the information on dynamic processes:

(9) $$G(t)=\langle \Delta \omega (0)\cdot \Delta \omega (t)\rangle =G(0)\cdot g(t),$$

where the angle brackets mean the ensemble averaging. For isotropic random motion g(t) is:

(10) $$g(t)=g(t)=e^{-\left|t\right|/\tau_{c}},$$

where $\tau_{c}$ is the correlation time. Applying Fourier transform to g(t), one obtains the frequency-domain spectral relaxation function j(ω) that is used to describe NMR relaxation processes. In terms of j(ω), the dipole contribution to the spin–lattice relaxation time is:

(11) $$\frac{1}{T_{1}}=G\left(0\right)\cdot \left[\frac{1}{3}j\left(\omega_{0}\right)+\frac{4}{3}j\left(2\omega_{0}\right)\right],$$

where $\omega_{0}$ is the resonance frequency. The factor G(0) contains information on the mutual arrangement of nuclear spins.

For liquids, the temperature dependence of $T_{1}$ can be well described within the Bloembergen–Purcell–Pound (BPP) model [99] that assumes the correlation time $\tau_{c}$ obeys the Arrhenius law:

(12) $$\tau_{c}=\tau_{0}exp\left(\frac{E_{a}}{k_{B}T}\right),$$

where $E_{a}$ is the activation energy of nuclear motion, $k_{B}$ is the Boltzmann constant and $\tau_{0}$ is a pre-exponential factor. In this case the spectral density is:

(13) $$j(\omega )=\frac{2\tau_{c}}{1+{\left(\omega \tau_{c}\right)}^{2}}.$$

The BPP model suggests a V shape of the $T_{1}(1/T)$ dependence with a minimum at $\omega_{0}\tau_{c}\approx 1$ [77]. For slow spin motion, which is often the case for confined liquids, the minimum is shifted towards a high temperature and is often outside the accessible temperature range. To probe slow spin motion, a spin-locking technique (relaxation in the rotating frame) can be applied. The application of the locking pulse $\omega_{1}$ allows for the displacement of the minimum into the experimentally accessible temperature window. The spin–lattice relaxation in this case is characterized by $T_{1\rho }$, which can be written as follows [77]:

(14) $$\frac{1}{T_{1\rho }}=G\left(0\right)\cdot \left[\frac{1}{2}j\left(\omega_{1}\right)+\frac{5}{6}j\left(\omega_{0}\right)+\frac{1}{3}j\left(2\omega_{0}\right)\right].$$

Note that Equation (14) is obtained at the exact resonance condition and for $\omega_{1}\ll \omega_{0}$.

The dipole contribution to the spin–spin relaxation time within the BPP model can be expressed as follows:

(15) $$\frac{1}{T_{2}}=G\left(0\right)\cdot \left[\frac{1}{2}j\left(0\right)+\frac{5}{6}j\left(\omega_{0}\right)+\frac{1}{3}j\left(2\omega_{0}\right)\right].$$

To study molecular mobility, relaxation times are measured as a function of temperature (less often, as a function of frequency, for example, applying the field cycling techniques). By fitting the experimental data using Equations (11)–(15), one obtains parameters of molecular motion $\left\{E_{a},\tau_{c}\right\}$. However, for solids, very often the temperature behavior of nuclear spins differs from the simple BPP model. The main issues are the distribution of correlation times and/or activation energy [100,101,102], the exchange between different fractions of adsorbed liquids (for example, on the surface and inside the voids) [103,104,105], the contribution of ion currents [106,107,108] or the interaction with paramagnetic ions [109] if present.

### 3.2. NMR Diffusometry

As noted above, depending on the zeolite topology, zeolite voids form a system of 1D, 2D or 3D channels.

Let us denote the last exponential factor in Equations (5) and (6) as $S_{ND}(t_{m},\tau ,g)$. It accounts for the diffusional signal decay in the case of unrestricted diffusion in 3D space. Let us rewrite this factor for an SFG STE case as:

(16) $$S_{3D}=\exp\left(-kD\right),$$

with $k={\left(\gamma g\tau \right)}^{2}(2\tau /3+t_{m})$. For diffusion in randomly oriented capillaries (1D diffusion) or in thin films (2D diffusion), the signal attenuation can be written as follows [110]:

(17) $$S_{1D}=\int_{0}^{1}\exp\left(-kDx^{2}\right)dx,$$

(18) $$S_{2D}=\exp\left(-kD\right)\int_{0}^{1}\exp\left(kDx^{2}\right)dx.$$

In the limit kD≪1, the 1D and 2D attenuations are singly exponential and are equal to exp(−kD/3) and exp(−2kD/3), respectively. Hence, for correctly determining the diffusion coefficient, it is necessary to take into account the dimension of the space, in which diffusion occurs.

**Figure 6:**
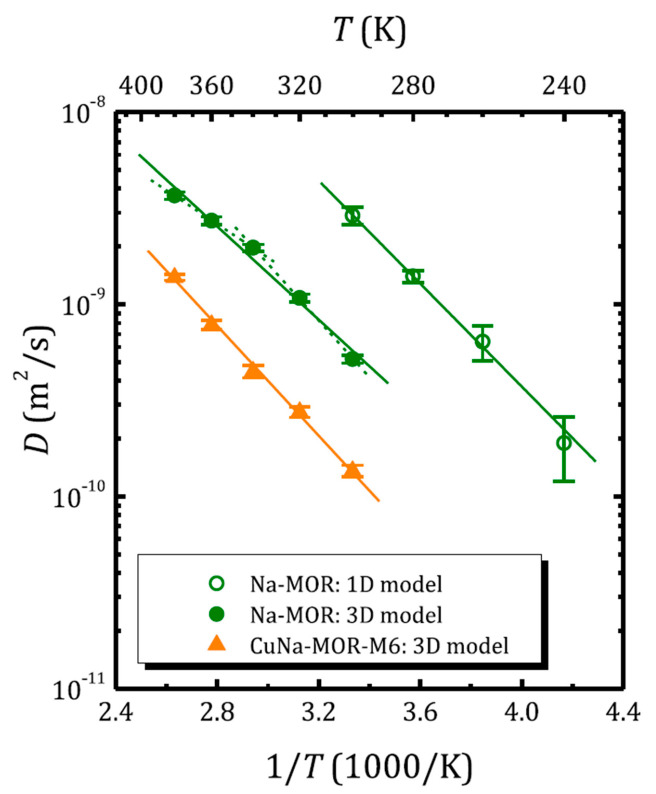
Water self-diffusion coefficient versus inverse temperature in sodium- and copper-exchanged mordenite (the ^1^H SFG STE experiment). The open and solid symbols correspond to the diffusion coefficient obtained applying the 1D and 3D diffusion model, respectively. Reproduced with permission from Krylova, E.A. et al. *Micropor. Mesopor. Mat.*; Elsevier Inc., 2018 [68].

Figure 6 shows the temperature dependence of the self-diffusion coefficient of water in zeolites with mordenite structure, as determined from ^1^H SFG STE [68]. The self-diffusion coefficient in sodium mordenite above and below 300 K was determined assuming intercrystallite (3D model; Equation (16)) and intracrystallite (1D model; Equation (17)) motion, respectively. Mordenite can be classified as a zeolite with a 2D network of voids: two types of channels are oriented along the *c*-axis and are interconnected through side pockets; see Figure 1. However, depending on the cationic form and the Si/Al ratio, the side pockets can be blocked by trapped molecules [68,111] or cations [112,113] and often mordenite is considered as a zeolite with a 1D channel system. Moreover, according to molecular dynamics (MD) simulation, at a low temperature, the water diffusion in voids of the fully silicated mordenite exhibits a 1D character, although, upon heating above 243 K, it passes from a 1D mode to 2D [114]. However, for the sodium form with Na^+^ located in the small mordenite channels, the side pockets can be partly blocked. Figure 6, therefore, clearly demonstrates how the model choice affects the magnitude of the self-diffusion coefficient derived from the same experiment.

The activation energy of water diffusion can be determined from the Arrhenius law:

(19) $$D=D_{0}exp\left(-\frac{E_{a}}{k_{B}T}\right)$$

and is less sensitive to the model choice.

**Figure 7:**
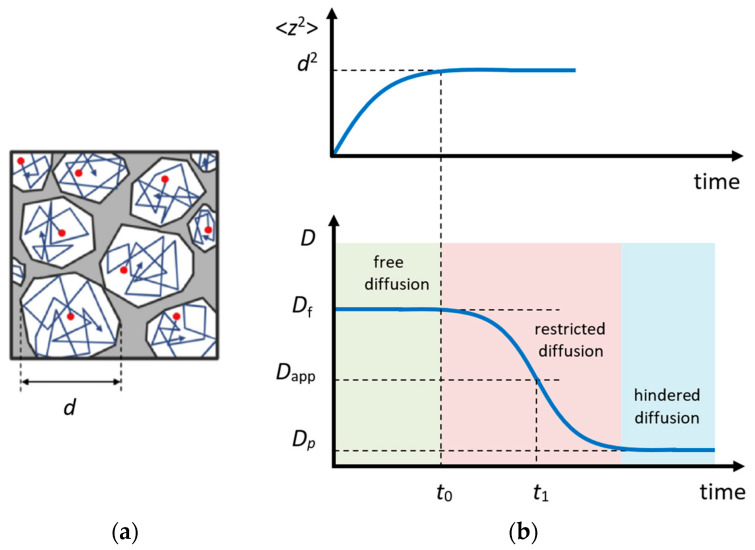
Restricted diffusion model: (**a**) motion of particles in a restricted area; (**b**) the mean square displacement of the particle in a restricted area (above) and the apparent diffusion coefficient (below) depending on time. Adapted with permission from Shelyapina M.G. et al. *Int. J. Hydrogen Energy*; Hydrogen Energy Publications, LLC. Published by Elsevier Ltd., 2015 [124].

In the experiment, the diffusional displacement of a particle is measured within a fixed time interval $t_{d}$ (in the STE method, $t_{d}=t_{m}$) that provides an exceptional opportunity to estimate the size and geometry of obstacles in the case of restricted diffusion, e.g., within porous media [115,116,117] or within crystallites [118,119]; Figure 7a. More information on this issue can be found in Refs. [116,120,121,122].

According to the Einstein equation that relates diffusion and mobility of a particle, in the case of free diffusion in a 3D space, the mean square displacement $\langle r^{2}\rangle$ of the observed particle depends on time as follows:

(20) $$\langle r^{2}\rangle =6Dt,$$

where the diffusion coefficient takes a value averaged over the time interval t. If, on a shorter time scale <t, the diffusion coefficient of the observed particle (or the mechanism of motion) changes, the apparent diffusion coefficient ($D_{app}$) will differ from the one measured on a short time scale. Such a diffusion is called restricted or anomalous (opposite to the normal Einstein diffusion) [120,123].

The character of the $D_{app}(t_{d})$ dependence is related to the shape of the diffusion area; however, a qualitative analysis can be conducted using a simple interpolation expression [122]:

(21) $$D_{app}(t_{d})=\frac{D_{f}\left(1-\frac{4}{9}\sqrt{\pi }\right)S}{V}\sqrt{D_{f}t_{d}},$$

where $D_{f}$ is the free diffusion coefficient and S/V is the surface-to-volume ratio of a cavity. Hence, by measuring $D_{app}(t_{d})$, one can estimate the effective size of the limited diffusion area.

For partially permeable pores, the $D_{app}(t_{d})$ dependence has a form shown in Figure 7b. In this case, the permeability of pore networks *P* can be estimated using a simple equation [125]:

(22) $$\frac{1}{D_{p}}=\frac{1}{D_{f}}+\frac{1}{Pd},$$

where d is the size of restricted area, which can be determined from Equation (21), and $D_{p}$ is the hindered self-diffusion coefficient in a region, where the total average of intrapore diffusion is achieved due to the permeability of the pore wall [116].

**Figure 8:**
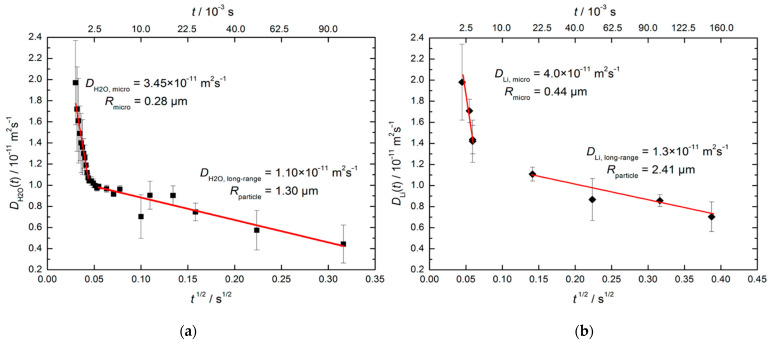
Effective diffusivities of water at 298 K (**a**) and of Li^+^ at 373 K (**b**) in hydrated zeolite Li-LSX. The straight lines show the fit of Equation (21) to the experimental data in the short- and long-time ranges. Reproduced with permission from Beckert, S. et al. *J. Phys. Chem. C*; American Chemical Society, 2013 [126].

Beckert et al. [126] applied SFG STE to study the time-dependent mean diffusion path lengths of H_2_O molecules (via ^1^H NMR) and Li^+^ cations (via ^7^Li NMR) in hydrated zeolite Li-LSX with faujasite (FAU) topology characterized by a 3D channel system; see Figure 1. They found the diffusivities decrease with increasing observation time due to transport resistances on the diffusion paths of water molecules and lithium cations. Drawing on the sample Scanning Electron Microscopy (SEM) microphotographs, possible issues of transport resistances are the outer surface of the zeolite particles and the boundary between the individual crystallites. Figure 8 shows water and Li^+^ diffusivities plotted versus $\sqrt{t_{d}}$. For both species, there are two regimes of the time dependence, “short-range” and “long-range”, that correlate with two types of resistance: “short-range” measurements refer to the intracrystalline diffusion and the resistance is related to barriers formed at the interfaces between the individual crystallites; “long-range” measurements refer to diffusion within the crystallite agglomerates, with transport resistances occurring at their external surface. Crystallite and particle sizes estimated using Equation (21) from ^1^H and ^7^Li measurements agree with each other and are consistent with the SEM images.

### 3.3. Water Dynamics in Zeolites

The partial substitution of Al for Si creates a negative charge in the zeolite framework compensated by cations and producing a strong electrostatic field that leads to vigorous interaction with polar molecules such as water [127,128,129]. Water is a promoter of many physical and chemical processes in zeolites, involving ion exchange that is carried out in aqueous solution. Water improves the efficiency of the ion-exchange process by co-ordinating cations and increasing their mobility [130] and affects the distribution and interaction of exchangeable cations with the zeolite framework [68,131,132]. In addition to high polarity, the water molecule is capable of forming up to four hydrogen bonds that result in a very high internal cohesiveness of bulk water [133]. In confined geometries, water molecules can interact both with pore surfaces and other water molecules, resulting in new elements of water structure due to the competing interactions. Zeolites provide clear examples of the effect of nanoconfinement on the water structure and behavior [45,134,135].

Water in zeolites have been extensively studied by various NMR methods for many years. Comprehensive reviews were conducted by Klinowskii [36] and Grey [35]. Here, we provide a short overview of recent results, focusing on relaxation studies.

**Figure 9:**
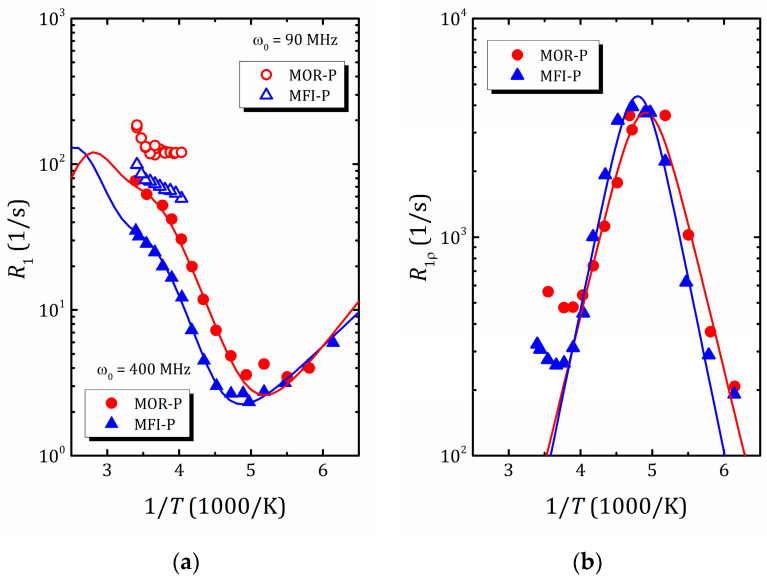
^1^H spin–lattice relaxation rate in laboratory frame (**a**) and rotating frame (**b**) versus inverse temperature in pillared mordenite (triangles) and pillared ZSM-5 (circles). Reproduced with permission from Shelyapina, M.G. et al. *Int. J. Mol. Sci.*; published by MDPI, Basel, Switzerland. Creative Common CC BY license, 2023 [45].

The temperature dependences of the proton spin–lattice relaxation time, $T_{1}$, allow us to determine the activation energy and correlation times, the characteristic parameters of water molecule motion. However, molecular motion, as a rule, includes both translational and reorientation components and, for liquids confined in zeolite voids, this dependence differs from the standard BPP model (where $T_{1}^{-1}(1/T)$ has a symmetric Λ shape) and can be rather intricate due to contributions from different types of motion and rearrangements of water structure. An example of such a complex temperature dependence of the proton spin–lattice relaxation rate ${R_{1}=T}_{1}^{-1}$ of water in mordenite and ZSM-5 zeolites with hierarchical porosity (2D zeolite lamella separated by amorphous SiO_2_ pillars preventing structure collapse and forming mesoporosity) [45] is shown in Figure 9a. As was mentioned above, for slow spin motions, the maximum of $T_{1}^{-1}(1/T)$ is shifted towards a high temperature and often lies beyond the accessible temperature range, which is the case for water confined in zeolites, as normally, above 300 K, zeolites start to desorb water. This further complicates the task of finding the parameters of molecular motion and, to determine them, the use of auxiliary tools, such as spin-locking [45,108] (that allows for the shifting of the maximum of the $T_{1\rho }^{-1}(1/T)$ dependence towards the desirable temperature window; see Figure 9b), NMR diffusometry and NMR spectroscopy (motional narrowing the spectral line) [45,136], as well as molecular dynamics simulation [114,137,138], is very helpful. The latter makes it possible to qualitatively estimate the correlation times for various types of motion that can be used to narrow down the search range for parameters of the model when fitting experimental data within expressions like Equations (11)–(15).

The activation energy values for various types of water motion in microporous and mesoporous zeolites, determined by various NMR methods, are listed in Table 1. It should be noted that, for confined water, the Arrhenius law (Equation (12)) does not hold at low temperatures, and the activation energy depends on temperature; therefore, the temperature ranges in which the studies were performed are specified in Table 1.

Paczwa et al. [139] combining ^1^H $T_{1}$ and $T_{1\rho }$ studied water mobility in natural natrolite from Khibiny deposit (Kola Peninsula, Russia) in a temperature range from 190 to 400 K. The natrolite structure contains two types of narrow channels (see Table 1) running parallel and perpendicular to the *c*-axis. As soon as the sizes of the channels are comparable with the diameter of water molecules (approximately 2.8 Å), the path and mechanism of water diffusion in natrolite are not evident. The proton relaxation studies suggested 2D water diffusion above 250 K with different activation energy. An additional contribution to the spin–lattice relaxation at low temperatures the authors associated with a contribution from paramagnetic impurities that commonly present in natural zeolites [4,12]. However, according to other studies, a flattening of the low-temperature branch of the $T_{1}(1/T)$ curve can be also associated with the translational motion of charged particles [108,124,140] or rotational motion of confined water molecules [114,141].

**Table 1:** Activation energy of intracrystalline water motion in micro- and mesoporous zeolites with different zeolite framework types as derived from various NMR experiments.

Zeolite	Si/Al	Micropore Size ^1^ (Å)	Mesopore Size (Å)	NMR Experiment	Temperature Range (K)	Type of Motion	Ea (kJ/mol)	Ref.
Natrolite (natural)	1.5	2.5 × 4.1 2.6 × 3.9	−	^1^H *T*_1_	190–400	Translational ‖ [001]	28	[139]
				^1^H *T*_1ρ_	220–380	Translational ⊥ [001]	37.3	[139]
Na,Ca-mordenite (natural)	5	7.0 × 6.5 3.4 × 4.8	−	^1^H *T*_1_	96–351	Translational ‖ [001]	20	[142]
				^1^H *T*_1ρ_	96–351	Translational	30	[142]
				^27^Al *T*_1_	200–365	Translational ‖ [001]	21.6	[143]
				^23^Na *T*_1_	200–365	Translational ‖ [001]	22.7	[143]
Na-mordenite	5.87	7.0 × 6.5	−	^1^H SFG	240–300	Translational ‖ [001]	25.6	[68]
Faujasite-NaX	~1.18 ^2^	7.4 × 7.4	−	^1^H PFG	254–353	Translational ‖ [111]	18.7	[66]
Faujasite-HY	2.39	7.4 × 7.4	−	^1^H *T*_1_	293–873	Translational ‖ [111]	25.6	[144]
Mesoporous NaA	NR ^3^	4.1 × 4.1	5.0	^1^H PFG	250–310	Translational	56.2	[66]
Pillared mordenite	8.4 ^4^	7.0 × 6.5	4.0	^1^H *T*_1_	173–293	Translational	23.6	[141]
				^1^H *T*_1_	173–293	Rotational	12	[45]
				^1^H *T*_1_	173–293	Freezing	29	[45]
				^1^H *T*_1ρ_	173–293	Freezing	28.9	[45]
Pillared ZSM-5	8.8 ^4^	0.55	4.4	^1^H *T*_1_	173–291	Translational	26.0	[45]
				^1^H *T*_1_	173–291	Rotational	9	[45]
				^1^H *T*_1_	173–291	Freezing	30	[45]
				^1^H *T*_1ρ_	173–291	Freezing	30.7	[45]

^1^ The size of micropores (channels) was taken from Ref. [1]. ^2^ Not reported; the Si/Al ratio was taken from Ref. [1]. ^3^ Not reported. ^4^ Data are taken from Ref. [38] for lamellar zeolites before pillaring with SiO_2_.

The same group [142] reported on the proton relaxation study of translational motion in natural mordenite from Nidym, Siberia. Two essentially different values of the activation energy of water motion in mordenite determined from $T_{1}$ and $T_{1\rho }$ temperature dependencies, 20 and 30 kJ/mol, respectively, the authors related with translational diffusion in larger and narrower mordenite channels [142]. However, the latter can be also associated with the translational motion through the side pockets, as predicted by molecular dynamics simulation [114]. Sergeev et al. showed that water mobility can be indirectly evaluated from spin–lattice relaxation of quadrupole ^27^Al and ^23^Na nuclei consisting zeolite that is governed by quadrupole coupling, which, in its turn, is modulated by translational motion of water molecules [143], wherein different methods, including diffusometry [68], give close activation energies of translational motion of water in mordenite channels, about 23 kJ/mol.

Zeolites with faujasite topology (zeolite X, zeolite Y and ultrastable zeolite Y (USY)) exhibit the most open framework of all natural zeolites; voids fill about half of the unit cell space; see Figure 1. Wide intersecting channels are formed parallel to the <111> directions of the cubic cell. Water molecules reside predominantly in the large cavities. In zeolites of the faujasite type, Si/Al ranges from 1.2 to 1.5 for X and from 1.5 to 2.5 for Y. The positions of Al and, hence, charge-compensating cations are also governed by the Si/Al ratio: in zeolites X in 6-MR rings, Al atoms are located in meta-positions, while, in zeolites Y, they are in para-positions. All this affects the water mobility confined in faujasite voids.

Katsiotis et al. [144] applied ^1^H NMR relaxation to study protonated zeolite Y. They found that $T_{1}$ displays a typical temperature dependence with a minimum at 423 K that can be fitted within the BPP model, providing a nominal activation energy $E_{a}$ = 25.6 kJ/mol; however, the temperature dependence of the spin–spin relaxation time $T_{2}$, which, instead of increasing with temperature rising, rapidly decreases up to 423 K and, after, remains constant, suggests a more complex mechanism. The authors suppose that, as intracage water dissociates by heating, proton motion mediated through the hydroxonium H_3_O^+^ vehicle mechanism becomes restricted, slowing down the relaxation behavior. On the other hand, the increase in the $T_{1}/T_{2}$ ratio means the enhancement of bonding of the proton with framework Al.

Recent studies of water mobility in hierarchical zeolites [45,141] confirmed that water in such a complex nanoconfinement (two-dimensional zeolite lamellae separated by amorphous SiO_2_) experiences complex temperature behavior. Two studied systems, pillared mordenite and pillared ZSM-5, show rather similar results. The temperature dependence of ^1^H $T_{1}$, see Figure 9a, suggests the presence of three different processes: freezing, fast rotation and translational motion of water. According to the ^1^H NMR spectral line narrowing, the water freezing occurs near 180 K [45], which, by applying the Waugh–Fedin expression [136], provides an estimate of the activation energy of about 30 kJ/mol. The activation energy of water rotational motion was found between 9 and 12 kJ/mol (depending on the zeolite framework topology), which is lower than in bulk water, and can be related with a low water density in mesopores. For translational motion, it was found to be 23.6 and 26.0 kJ/mol for pillared mordenite and ZSM-5, respectively, which is very close to the values in microporous zeolites but essentially lower than for water in mesoporous silicas or zeolites with a similar size of mesopores but with disordered mesoporosity; see Table 1. Similar to silicas in pillared zeolites, only a part of water interacts with silanol groups on SiO_2_ pillars, forming mesoporosity. This water fraction is characterized by fast rotational but slow translational motion. The rest of water is involved in translational motion in ordered zeolite micropores. All this allowed the authors to suppose that the diffusion process in zeolites with a hierarchically organized pore structure is affected both by mesoporosity and by the mutual arrangement of meso- and micropores; moreover, the translational motion of water molecules is determined mainly by the zeolite micropores [45].

## 4. Probe of Acidity: 2D $T_{1}$–$T_{2}$ Correlation Maps

Over the past decade, there has been a rapid development in the use of NMR relaxation as a method for determining surface affinity and adsorbate behavior in catalytically active porous media, including zeolites.

For confined liquids, where the exchange that molecules experience between the surface layer of thickness ε cannot be neglected, the observed nuclear spin relaxation rates $T_{1,2}^{-1}$ can be represented as follows [145,146,147]:

(23) $$\frac{1}{T_{1,2}}\approx \frac{1}{T_{1,2}^{bulk}}+\frac{1}{\frac{d_{p}}{2\alpha \rho_{1,2}}+\frac{d_{p}^{2}}{8\alpha D}}=\frac{1}{T_{1,2}^{bulk}}+\frac{2\alpha \rho_{1,2}}{d_{p}}\cdot \frac{1}{1+\frac{\rho_{1,2}d_{p}}{8\alpha D}},$$

where $\rho_{1,2}=\varepsilon \cdot {{(T}_{1,2}^{surf})}^{-1}$ is the surface relaxivity, $T_{1,2}^{bulk}$ and $T_{1,2}^{surf}$ are the corresponding relaxation times for the bulk liquid and molecules adsorbed on the surface, D is the self-diffusion coefficient of the unrestricted bulk liquid,$\;d_{p}$ is the pore diameter and α is a parameter that characterizes the pore shape (α= 1, 2 or 3 for planar, cylindrical or spherical pores, respectively). Note that Equation (23) was obtained for long times t and under conditions of the fast exchange with a rate w: $t\gg T_{1,2}^{surf}\gg 1/w$. Let us consider two extreme cases. In the case of the diffusion-limited relaxation, $4D/d_{p}\ll \rho_{1,2}$, Equation (23) can be simplified:

(24) $$\frac{1}{T_{1,2}}\approx \frac{1}{T_{1,2}^{bulk}}+\frac{8\alpha D}{d_{p}^{2}}.$$

In the case of the surface-limited relaxation, when $4D/d_{p}\gg \rho_{1,2}$, it can be written as:

(25) $$\frac{1}{T_{1,2}}\approx \frac{1}{T_{1,2}^{bulk}}+\frac{2\alpha \rho_{1,2}}{d_{p}},$$

and, for spherical pores of volume *V* and surface *S*, one obtains the generally applied expression:

(26) $$\frac{1}{T_{1,2}}\approx \frac{1}{T_{1,2}^{bulk}}+\rho_{1,2}\frac{S}{V}.$$

It means that, for the confined geometry, there is a linear correlation between the observed relaxation rates $T_{1,2}$ and $d_{p}$ or $d_{p}^{2}$, depending on the relaxation limiting conditions. On the one hand, it opens opportunities to use NMR relaxation as a tool to probe the morphology of porous media, as will be discussed further. On the other hand, the surface relaxivities of molecules adsorbed on microporous materials are a measure for host–guest interactions. For instance, relaxation measurements were used to investigate confinement effects on CO_2_ and CH_4_ admission in ZIF-8 metal organic framework and ZSM-58 zeolite [148]. By plotting $T_{1}^{-1}$ of ^1^H or ^13^C as a function of inverse gas density, it was found that, for ZIF-8, the surface relaxation is independent of the adsorbed gas density, while, in ZSM-58 with small eight-ring windows, the density dependence of the relaxation rates of adsorbed methane indicates stronger host–guest interactions at low gas loadings.

The ratio $T_{1}/T_{2}$ has been found to be very worthwhile to prove molecular mobility at the solid/liquid interface. Due to the decrease in rotational and translational molecular mobility upon adsorption on the surface, the surface relaxation times became shorter, $T_{1,2}^{bulk}\gg T_{1,2}^{surf}$. Then, the ratio of the surface spin–lattice and spin–spin relaxation times is:

(27) $$\frac{T_{1}}{T_{2}}\approx \frac{\rho_{1}}{\rho_{2}}=\frac{T_{2}^{surf}}{T_{1}^{surf}}.$$

This ratio is sensitive to surface affinity [70] and can be used as a noninvasive method for determining surface acidity affinity of porous zeolites [71]. It was shown that the $T_{1}/T_{2}$ ratio correlates with the desorption energy of liquids absorbed into zeolite matrix [71,72].

In studies of molecular structures and dynamics, modern NMR spectroscopy relies on multidimensional correlations. Likewise, the multidimensional correlation functions of $T_{1}$ and $T_{2}$ can also be used to identify molecular species and to probe their dynamics. For this purpose, $T_{1}$–$T_{2}$ correlation data are acquired by applying a 2D NMR pulse sequence, as shown in Figure 5a. The resulting maps evidence the relationship between the ratio of relaxation times of the adsorbed liquid and the properties of the surface of a porous medium.

**Figure 10:**
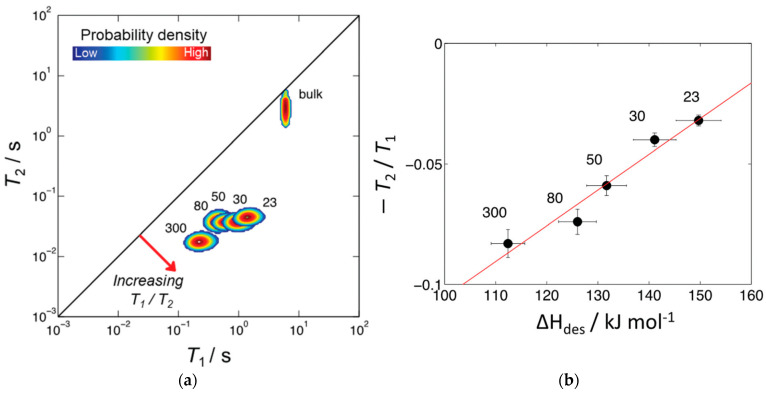
(**a**) ^1^H $T_{1}$–$T_{2}$ correlation plots for pyridine in HZSM-5 with varying SiO_2_/Al_2_O_3_ ratios. The diagonal line indicates the parity ratio $T_{1}/T_{2}=1$; bulk pyridine data are also shown; (**b**) $-T_{1}/T_{2}$ plotted versus enthalpy of pyridine adsorption; the red line shows a linear fit. SiO_2_/Al_2_O_3_ values are indicated next to each correlation peak or point. Reproduced with permission from Robinson, N. et al. *Phys. Chem. Chem. Phys.*; published by the Royal Society of Chemistry. Creative Common CC BY license, 2021 [71].

Robinson et al. [71] proposed the $T_{1}/T_{2}$ ratio as a probe for zeolite acidity. Figure 10a shows the 2D $T_{1}$–$T_{2}$ correlation map for pyridine adsorbed in the pores of microporous ZSM-5 zeolites with different SiO_2_/Al_2_O_3_ ratios. Correlation peaks indicate the relative probability density of each pyridine/zeolite system. Increasing $T_{1}/T_{2}$ can be interpreted as indicative of surface host–guest interaction strength. The authors suggested that the position of these peaks is conditioned by the relative surface affinities of pyridine within zeolite matrices. Indeed, for the studied series of ZSM-5 zeolites, the $T_{1}/T_{2}$ ratio plotted versus enthalpy of pyridine desorption $\Delta H_{des}$ shows a linear correlation between these two parameters. $\Delta H_{des}$, in its turn, increases with decreasing SiO_2_/Al_2_O_3_ (increasing the number of Brønsted acid sites); see Figure 10b.

Further relaxation studies of adsorbed water in ZSM-5 and chabazite conducted by d’Agostino et al. [149] confirmed that the $T_{1}/T_{2}$ ratio can be used as an indicator of host–guest surface interactions within a given zeolite framework that are related with Brønsted acidity. However, the authors note that, when comparing zeolite frameworks with different pore sizes, besides host–guest interactions, it is necessary to take into account the confinement effects. A more dramatic increase in $T_{1}/T_{2}$ with increasing Al content for chabazite, which is characterized by a smaller pore size (3.8 × 3.8 Å) compared to ZSM-5 (sinusoidal channels: 5.3 × 5.6 Å; straight channels: 5.5 × 5.1 Å), indicates reduced mobility of water molecules (kinetic diameter: 2.7 Å) due to an increased interaction with the chabazite surface [149].

The guest–host interaction for toluene adsorbed into H-Beta and Pd-modified H-Beta zeolites was probed by NMR relaxation by Zue et al. [150]. For both compounds, the dealumination results in an increase in the $T_{1}/T_{2}$ ratio, confirming the enhanced interaction between toluene molecules and the zeolite surface. In addition, the Pt loading leads to a considerable increase in $T_{1}/T_{2}$ that authors interpreted as an ability of dealuminated Pd-modified H-Beta zeolites to interact with toluene, providing partial oxidation of the adsorbed toluene to benzyl alcohol facilitated by the zeolite hydroxyls.

Forster et al. [151] applied NMR relaxation measurements to evaluate the effect of the reaction solvent (water, methanol and ethanol) upon the catalytic activity of Sn- and Ga-doped zeolite Y for the isomerization of glucose to fructose. NMR relaxation suggested that the lack of catalytic activity in water is due to the strong adsorption of water molecules within the zeolite pores, which makes the Lewis acid sites active for the sugar isomerization inaccessible for reactants. Ethanol, compared to methanol, is more easily adsorbed in zeolite pores, where Lewis acid sites transform glucose to fructose. Ethanol being retained in the pores prevents solvated fructose from further reaction on Brønsted acid sites situated outside of the pore space.

Besides probing surface acidity, 2D $T_{1}$–$T_{2}$ measurements can be used for mineral deposit analysis, in particular, such complex porous media as clay minerals containing multiple components [152,153,154]. While 1D *T*_2_ distribution recorded at low field (commonly used frequencies are 2–20 MHz for ^1^H) now is routine for rock core analysis [155], 2D $T_{1}$–$T_{2}$ correlation maps provide more comprehensive data on fluid saturation and its states [152]. So far, to our knowledge, no such study has been conducted for natural zeolites.

## 5. NMR Cryoporometry

In recent years, NMR cryoporometry (NMRC) has been increasingly used to study the pore size distribution on a scale from sub-nanometers to several micrometers. Comprehensive reviews that discover the main principles of this method can be found in Refs. [146,156].

The main idea of NMR cryoporometry is to detect the temperature shift of phase transitions of a matter due to confinement within the porous matrix. These shifts can be interpreted in terms of pore geometry and, consequently, information can be obtained about pore sizes and their distribution and, in favorable cases, about the shape of the pores. As compared to gas adsorption technics that use the Kelvin equation (constant temperature), NMR cryoporometry relies on the Gibbs–Thomson equation (constant pressure). So, for example, for a case of cylindrical pores with radius r, the shift of the melting point $T_{m}$ can be expressed as [157]:

(28) $$\Delta T_{m}\equiv T_{m}-T^{0}=-\frac{4\sigma_{sl}T_{m}}{r\Delta H_{f}\rho_{s}},$$

where $\sigma_{sl}$ is the surface energy of the solid–liquid interface, $\Delta H_{f}$ is the bulk enthalpy of fusion and $\rho_{s}$ is the density of the solid. In a similar way, the shift in the freezing point can be defined as $\Delta T_{f}\equiv T_{f}-T^{0}$. This equation can be simplified for specific cases. For example, for cylindrical pores in the large pore limit (>10 nm), it is possible to obtain the following simple expressions [156]:

(29) $$\begin{array}{c} \Delta T_{m}\equiv -\frac{K_{c}}{r},\\ \Delta T_{f}\equiv -\frac{{2K}_{c}}{r}. \end{array}$$

**Figure 11:**
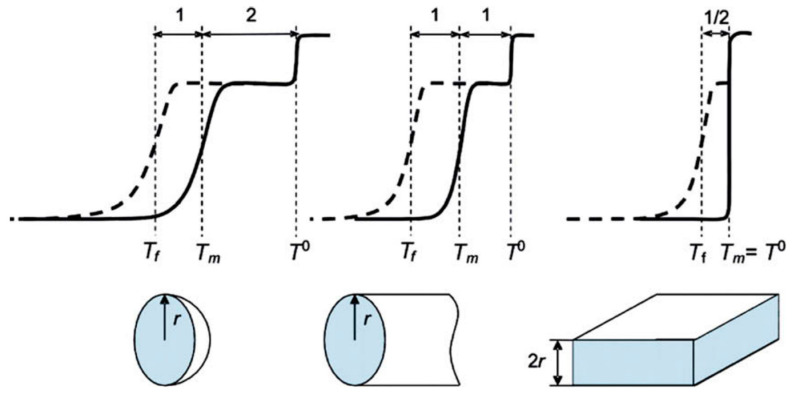
Scratch to illustrate relationship between the freezing–melting hysteresis form and model pore geometries in the large pore limit (>10 nm); dashed and solid lines correspond to freezing and melting, respectively. Reproduced with permission from Petrov, O.V., Furó, I. *Prog. Nucl. Magn. Reson. Spectrosc.*; Elsevier B.V. 2008, [156].

For other models of pore shapes, the Gibbs–Thomson relationship is provided in Table 2 and illustrated in Figure 11. As soon as the $\Delta T_{m}/\Delta T_{f}$ ratio is sensitive to the pore shape, it can be used to assess the pore geometry.

In an NMR cryoporometry experiment, the signal intensity is proportional to the pore volume v(x). If the pores are filled with liquid, its melting temperature $T_{m}$ is related to the pore size distribution. Hence, the measurement of pore size distributions is accessed by differentiating and remapping the melting curve data using the following expression [158]:

(30) $$\frac{dv(x)}{dx}=\frac{K_{c}}{x^{2}}\frac{dv}{dT_{m}(x)}.$$

So, for NMR cryoporometry measurements, a porous material with an adsorbed probe liquid is cooled until all the liquid in the pores is frozen; after that, the sample is slowly heated. Hence, to determine a pore size distribution, it is enough to record the intensity of the liquid signal as a function of temperature.

Normally, NMR cryoporometry detects ^1^H $T_{2}$ signals from adsorbed substances (typically water or organic molecules). The solid phase is characterized by short spin–spin relaxation times $T_{2}$ of the order of microseconds, while liquid phases have characteristic $T_{2}$ values from milliseconds to seconds. This usually makes it easy to distinguish signals from solid and liquid fractions, although there may be exceptions in the case of a soft plastic crystalline phase with a relatively long spin–spin relaxation time, as in the case of cyclohexane. The temperature behavior of $T_{2}$ of the liquid fraction can be obtained by measuring an echo train using the CPMG pulse sequence.

**Figure 12:**
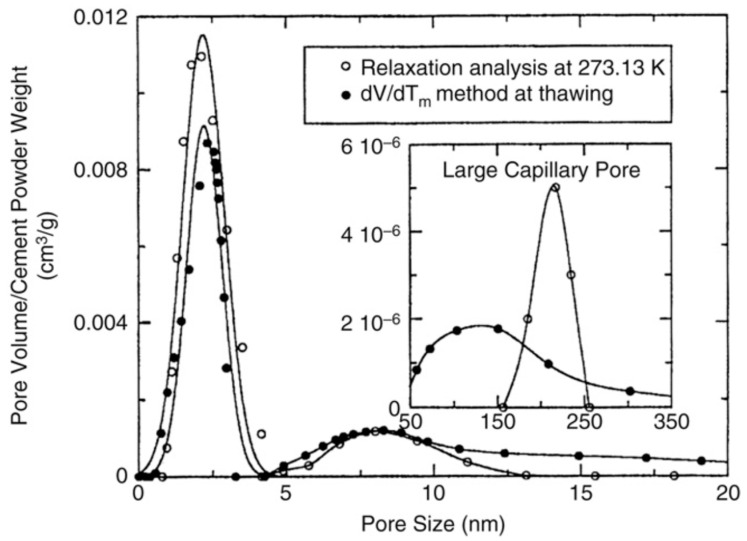
Pore size distribution for a cement paste determined by NMR cryoporometry (solid symbols) and NMR relaxometry (open symbols). Reproduced with permission from Jehng, J.Y. et al. *Magn. Reson. Imaging*; Elsevier Science Inc., 1996 [159].

Figure 12 shows pore size distribution in cement paste determined by NMR cryoporometry and NMR relaxometry [159]; for both cases, water was used as adsorbate. These two methods exhibit good agreement for micro- and mesopores but, for macropores, a certain discrepancy between two methods is observed.

The main advantages of NMR cryoporometry over standard gas adsorption and thermo-porosimetry are a wide variety of substances that can be used as a probe liquid and that predrying of the samples is not essential and, hence, it can be used for those materials whose pore structure would suffer if predried (wet clays and hydrogels). For zeolites, the latter is not so crucial, and traditional methods are more in demand. 

Webber et al. [73] reported the results of NMR cryoporometry to probe the micro- and mesopore size distributions in USY zeolites compared to Barrett–Joyner–Halenda (BJH) model for N_2_ adsorption to construct a pore size distribution. They found that both methods account for the mesopore volume, while the micropore volume is underestimated relative to the *t*-plot method. However, the pore size distribution obtained within NMR cryoporometry is better resolved in a small mesopore range compared to a smoothed one derived using BJH.

Recently, Fleury et al. [74] reported results of the pore size distribution in microporous and partly mesoporous UZY zeolite probed by NMR cryoporometry and mercury intrusion experiments. Whereas both technics yield the same average meso- and macropore size, only NMR cryoporometry detects the larger macropores.

## 6. Pore Connectivity: 2D $T_{2}$–$T_{2}$ Exchange Maps

Two-dimensional relaxation exchange NMR experiments are very helpful to evidence molecular transport between different relaxation environments and can be used to probe connectivity in systems with complex hierarchical porosity [95]. Relaxation exchange experiments help to evidence diffusive coupling between two relaxation environments [76].

The magnetization recorded in 2D relaxation exchange NMR is described by Equation (8). By applying 2D inverse Laplace transform, one can determine the probabilities $F(T_{2}^{A},T_{2}^{B})$.

**Figure 13:**
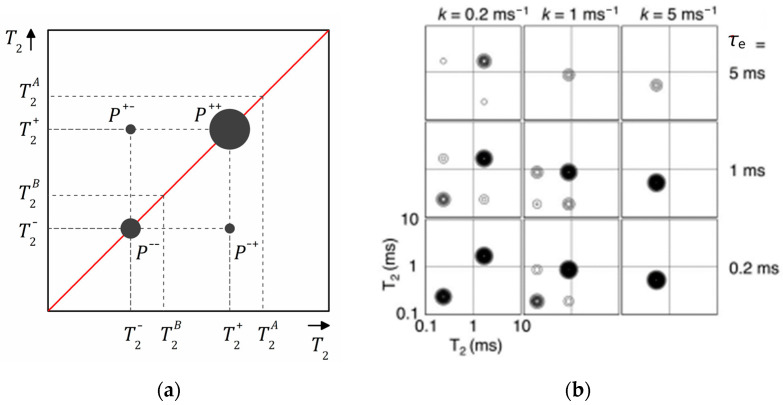
(**a**) A schematic $T_{2}$–$T_{2}$ map showing diagonal and off-diagonal peaks at a certain exchange time $\tau_{e}$; (**b**) calculated $T_{2}$–$T_{2}$ maps for slow (**left**), intermediate (**middle**) and fast (**right**) exchange rates k and short (**bottom**), intermediate (**middle**) and long exchange time $\tau_{e}$. Reproduced with permission from Monteilhet, L. et al. *Phys. Rev. E*; The American Physical Society, 2006 [161].

Let us consider two reservoirs of magnetization, $M_{A}$ and $M_{B}$, that correspond to nuclei in pores of different sizes, for example, micro- and mesopores. And let us assume that there is an exchange between these two reservoirs. In this case, the relaxation of the magnetization is governed by the system of coupled differential equations [160]:

(31) $$\begin{array}{c} \frac{dM_{A}}{dt}=-k_{A}M_{A}+k_{B}M_{B}+\frac{1}{T_{1,2}^{A}}\left({M_{A}^{eq}-M}_{A}\right),\\ \frac{dM_{B}}{dt}=-k_{B}M_{B}+k_{A}M_{A}+\frac{1}{T_{1,2}^{B}}\left({M_{B}^{eq}-M}_{B}\right), \end{array}$$

where $k_{A}$ and $k_{B}$ are the exchange rates from *A* to *B* and from *B* to *A* and $M_{A,B}^{eq}$ is the equilibrium magnetization for each reservoir, which is equal to zero for spin–spin relaxation. The analytical solution of this system of equations can be found in Ref. [161]. The obtained 2D $T_{2}$–$T_{2}$ map is schematically shown in Figure 13a. Off-diagonal peaks at an exchange time $\tau_{e}$ indicate diffusive exchange between two reservoirs *A* and *B* (relaxation populations). The diagonal peaks appear at $T_{2}^{+}$ and $T_{2}^{-}$ that differ from the “real” $T_{2}^{A}$ and $T_{2}^{B}$ relaxation times if there is an exchange. Recording a series of $T_{2}$–$T_{2}$ maps at various exchange times $\tau_{e}$, one can plot the amplitudes of the diagonal $P^{++}$, $P^{--}$ and off-diagonal $P^{+-}$, $P^{-+}$ peaks as a function of $\tau_{e}$ and determine the exchange rates $k_{A}$ and $k_{B}$. The numerical expression of these amplitudes can be found in Ref. [161].

Figure 13b shows results of simulation of $T_{2}$–$T_{2}$ maps conducted by Monteilhet et al. [161] assuming a two-sites model and with $k_{A}=k_{A}=k$ (two reservoirs of equal size) within the above described model. It allows us to follow how the peak positions and intensities depend on the exchange rate k and the exchange time $\tau_{e}$. The simulation was conducted for $M_{A}^{eq}=M_{B}^{eq}=0.5$, $T_{2}^{A}=T_{1}^{A}/4=0.25$ ms and $T_{2}^{B}=T_{1}^{B}/4=2.5$ ms. For slow exchange and short exchange time (Figure 13b, bottom left), the 2D spectrum consists of two intensive diagonal peaks $P^{++}$ and $P^{--}$ of comparable amplitude close to ${(T}_{2}^{A},T_{2}^{A})$ and ${(T}_{2}^{B},T_{2}^{B})$ points on the 2D $T_{2}$–$T_{2}$ map (in the limit k→0 and $\tau_{e}\to 0$, the peaks are of equal amplitude and centered exactly on these positions). With the exchange rate increasing, the peaks shift along the diagonal, their intensities change and one becomes dominating and, simultaneously, off-diagonal peaks appear. In the case of fast exchange, only one diagonal peak corresponding to the average $T^{2}$ value is observed and off-diagonal peaks disappear. With the exchange time $\tau_{e}$ increasing, the total intensity of the peaks goes down due to spin–lattice relaxation losses. This is particularly true for the lower diagonal peaks (short $T_{2}$ and short $T_{1}$). However, the fraction of the intensity of the off-diagonal peaks increases.

In a real experiment, the integral peak intensities $P^{ij}$ (where i,j mean+or−) are determined at different exchange times and, within the described above model, the exchange rate k can be found by fitting the experimental data by the following the equation [93,162]:

(32) $$\frac{P^{+-}+P^{-+}}{P^{tot}}=A\left(1-e^{\frac{\tau_{e}}{k}}\right),$$

where $P^{tot}$ is the summed amplitude of all peaks and *A* characterizes the amount of molecules exchanged.

**Figure 14:**
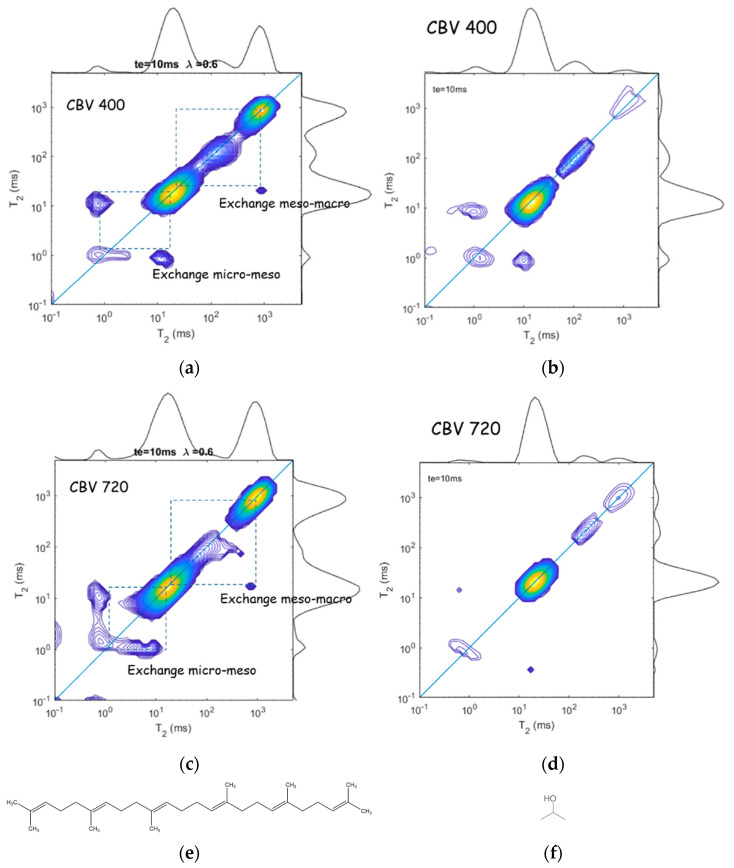
Relaxation exchange experiments with squalane (**a**,**c**) and 2-propanol (**b**,**d**) adsorbed by UZY zeolites with low (**a**,**c**) and high (**b**,**d**) mesoporosity. The off-diagonal amplitudes indicate the diffusive coupling between micro- and mesopores, as well as between meso- and macropores. (**e**,**f**) show squalane and 2-propanol molecules, respectively. Adopted with permission from Fleury, M. et al. *Micropor. Mesopor. Mat.*; Elsevier Inc., 2023 [74].

The above described NMR 2D $T_{2}$–$T_{2}$ exchange experiment provides additional information about the dynamics of liquids confined in different interconnected pores, which is especially useful for exploring systems with hierarchical porosity. Fleury at al. [74] applied 2D relaxation experiments with squalane and propanol to evaluate exchange rates between zeolite microporosity and surrounding mesopores in mesoporous USY zeolites; Figure 14. They studied two samples: CBV400 (a slightly dealuminated USY zeolite with a fairly low mesopore volume) and CBV720 (a strongly dealuminated form of USY, with a significantly higher internal mesoporosity and a higher external crystalline surface). Microporous USY zeolites belong to the framework type FAU, with 3D channels of 7.4 × 7.4 Å; see Figure 1. Squalane is the largest alkane molecule that, close to solid surfaces, can have a diameter of about 5 Å that allows it to penetrate into the micropores and to exchange between meso- and/or micropores. 2-propanol as a probe allows the porous network to be explored in another way: having a hydroxyl group, this molecule strongly interacts with the zeolite surface and, therefore, the surface diffusion mechanism becomes important.

The 2D $T_{1}$–$T_{2}$ maps for squalane indicate multiple $T_{1}/T_{2}$ ratios that can be attributed to macropores ($T_{1}/T_{2}=1$ means molecules very weakly interact with the zeolite surface and behave as in the bulk), to mesopores ($T_{1}/T_{2}$ = 4 indicates molecules interact with the zeolite surface), and to micropores ($T_{1}/T_{2}$ = 20 and 40 for CBV400 and CBV720, respectively, which is typical for pseudo-solid behavior when rotational motions are severely limited). Comparing these different pore fractions estimated from $T_{2}$ relaxation to the reference value measured by NMR cryoporometry ($T_{2}$ measurements essentially underestimate microporosity and overestimate mesoporosity), the authors made a conclusion about strong exchange between micro- and mesopores [74]. A stronger underestimation of microporosity from $T_{2}$ for a given molecule means a stronger connectivity between micro- and mesopores.

Relaxation exchange experiments provide direct evidence of diffusive coupling between two relaxation environments that can be used to probe pore connectivity [76]. Figure 14a,c show 2D $T_{2}$–$T_{2}$ maps for squalane confined in CBV400 and CBV720, respectively [74]. Symmetric off-diagonal peaks evidence diffusive coupling between the micro- and mesopores, while a single asymmetric peak points out diffusive coupling between meso- and macropores. Analysis of off-diagonal peak intensities as a function of the exchange time results in squalane molecule exchange between the micro- and mesopores with a timescale of about 6 ms for both zeolites but a larger amount of molecules exchanged for CBV720 that correlates with the data on microporosity.

For 2-propanol (Figure 14b,d), only exchange peaks between micro- and mesopores were observed. Analysis of the off-diagonal terms applying Equation (32) results in k = 2 and 4 ms for CBV400 and CBV720, respectively; however, the amount of exchanged 2-propanol molecules in CBV400 is one order of magnitude greater than in CBV720, which the authors associated with the high surface residence time of 2-propanol in micropores of CBV720 due to the strong interaction of polar molecules with the zeolites framework.

It should also be added that the two-sites model is normally used to analyze experimental data; however, it is not always applicable to real systems. A more carful theoretical study within the three-sites model conducted by Gao and Blümich [96] shows that (i) the exchange map can be asymmetric in the case of microscale vortex motion, (ii) the peaks shift in the apparent 2D two-site $T_{2}$–$T_{2}$ exchange maps, and (iii) there can be negative peak amplitudes (it can indicate that more molecules move from one site to another in the evolution or detection periods than in the mixing period).

Despite its visibility and great potential for the study of systems with complex pore organization, up to now, $T_{2}$–$T_{2}$ relaxation exchange NMR is little used for the study of zeolites. Nevertheless, in the last few years, a number of publications have appeared on the study of related systems. For instance, Elgersma et al. applied it to measure the liquid–solid mass transfer coefficient for water flowing through in packed beds of porous silica (or silica/titania) pellets [163,164]. The authors proposed this method for the screening and optimization of catalyst pellets and reactor operating conditions.

## 7. Conclusions

The main goal of this review was to demonstrate the great potential of NMR relaxation techniques to bring insight into various properties of zeolites and zeolite-based catalysts with complex pore architecture. In recent years, various low-field NMR relaxation methods along with fast field cycling and ultralow-field NMR have emerged as a complementary approach to traditional high-field NMR techniques. These methods result in not only cost saving but open up new opportunities.

One of the advantages of the NMR relaxation techniques described above is relatively modest requirements for the instrument base compared to solid-state NMR spectroscopy: they do not require high field for better resolution, fast rotation of the sample or special setup to prepare hyperpolarized ^129^Xe. Besides the traditional application to study the mobility of adsorbed molecules, both rotational and/or translational motion, by measuring the temperature dependences of relaxation times and self-diffusion coefficients, NMR relaxation provides access to surface acid sites and, by combining different NMR techniques, it is possible to obtain valuable information on textural properties of catalysts at different scales, which is of special interest for systems with hierarchical pore structure.

Although, generally, NMR relaxation gives rather integral characteristics as compared to multiple-quantum and 2D MAS NMR, in some cases, the information extracted is comparable and even surpasses that obtained by other methods. In particular, NMR cryoporometry disposes a wide variety of substances that can be used as a probe liquid and gives access to a larger scale of pore sizes compared to standard gas adsorption and thermo-porosimetry methods. However, despite its obvious advantages and a number of fascinating studies that have appeared in the last few years, NMR relaxation is still not in sufficient demand for probing porous systems and zeolites in particular. We hope that this review will expand the range of researchers who include this method among other useful tools for characterizing zeolites and zeolite-based materials with complex topology of voids and active sites on their surface.

## Figures and Tables

**Table 2:** The freezing and melting temperature shift $\Delta T_{f}$ and $\Delta T_{m}$, respectively, for various pore geometries in the limits of large pores [156].

Pore Shape	$|\Delta T_{f}|(K)$	$|\Delta T_{m}|(K)$
Sphere	${3K}_{c}/r$	${2K}_{c}/r$
Cylinder	${2K}_{c}/r$	$K_{c}/r$
Slit	$K_{c}/r$	0
